# Physical and chemical differences between one-stage and two-stage hydrothermal pretreated hardwood substrates for use in cellulosic ethanol production

**DOI:** 10.1186/s13068-016-0446-9

**Published:** 2016-02-03

**Authors:** Andrew Guilliams, Sivakumar Pattathil, Deidre Willies, Matt Richards, Yunqiao Pu, Sindhu Kandemkavil, Erin Wiswall

**Affiliations:** Mascoma LLC, 67 Etna Road, Lebanon, NH 03766 USA; Lallemand, 8215 Beachwood Road, Baltimore, MD 21222 USA; CCRC, 315 Riverbend Road, Athens, GA 30602 USA; Georgia Tech Renewable Bioproducts Institute, 500 10th Street NW, Atlanta, GA 230332 USA

**Keywords:** Biofuels, Pretreatment, Glycome profiling, Cellulosic ethanol, Hardwood fermentation

## Abstract

**Background:**

There are many different types of pretreatment carried out to prepare cellulosic substrates for fermentation. In this study, one- and two-stage hydrothermal pretreatment were carried out to determine their effects on subsequent fermentations. The two substrates were found to behave differently during fermentation. The two substrates were then characterized using physical and chemical parameters.

**Results:**

The one-stage substrate was found to have higher carbohydrate content and lower lignin content. It exhibited a higher level of viscosity, a larger settled volume, and a slower settling time than the two-stage substrate. It also showed higher polarity and reduced crystallinity. Glycome profiling showed physical differences between the two substrates, specifically pointing toward higher levels of pectin and hemicellulose in the one-stage substrate (MS1112) as compared to the two-stage substrate (MS1107).

**Conclusions:**

We hypothesize that these physical and chemical differences between the substrates contribute to the differences seen during fermentation including: ethanol yield, ethanol titer, fermentation rate, fermentation completion time, mixing, and substrate solubilization. These findings can be used in optimizing pretreatment parameters to maximize ethanol conversion and overall process yield for hardwood substrates.

**Electronic supplementary material:**

The online version of this article (doi:10.1186/s13068-016-0446-9) contains supplementary material, which is available to authorized users.

## Background

Conversion of sugars in cellulosic feedstocks, an abundant and potentially sustainable source of organic complex molecules, into fuels and chemicals has drawn the interest of scientists and industry for decades [1–4]. The breakdown of these complex feedstocks into monomeric sugars that can then be readily converted into fermentation products, remains a key barrier for their usage [5]. Many organisms produce enzymatic systems that can attack cellulosic biomass and break it down to its constituent monomeric sugar molecules [6–8], with the most widely used industrial system being that of aerobic fungi like *Trichoderma reesei*. To prepare the feedstock for enzymatic attack energy intensive pretreatments, often with the use of harsh chemicals, are used [9–13]. These include dilute acid, AFEX™, and hydrothermal pretreatment, among others.

Different methods of pretreatment have been tried on different feedstocks with varied success [9–11, 14]. The materials that result from these pretreatments often behave differently during enzymatic hydrolysis and/or fermentation [9–11, 15, 16]. For example, single-stage and two-stage hydrothermal pretreatment produce differences in lignin content, cellulose crystallinity, hemicellulose content, and porosity of the substrate depending on the length and severity of the pretreatment steps [16–19]. Cui et al. [9] have attempted to quantify the various differences pretreatment methods can have on enzymatic saccharification and cellulose structure. Chemicals and energy are expensive and a method of pretreatment must be found that liberates as much cellulose as possible while also remaining cost effective [15]. The optimization of yield and cost is a necessary part of ensuring the process will be commercially viable. During pretreatment, a hemicellulose fraction is also liberated which can be fermented to ethanol [20]. Part of the optimization is maximizing the yield of the hemicellulose fraction as well as the cellulose fraction. It is beneficial to subject the pretreated material to a washing step before saccharification to remove this hemicellulose stream [11, 15]. The more severe the pretreatment step (as determined by Eq. 1), the more sugar is available for saccharification, but only to a point. Hydrothermal pretreatment is generally classified by severity factor which is explained in Eq. 1 by Overend and Chornet. Upon the use of very severe pretreatment regimens, the sugars are converted instead to degradation products that are not suitable for fermentation. The most common degradation products are hydroxymethylfurfural (HMF) for hexose sugars and furfural for pentose sugars. This only serves to compound the optimization needed around pretreatment, although since more severe pretreatments generally are less cost effective, this imparts some basic limits on the range of pretreatments to explore. We chose to explore the higher than normal pretreatment severity factor in order to combat the recalcitrance of the hardwood feedstocks we have chosen to explore. This decision was also driven by testing many different severities of both one-stage and two-stage pretreatment conditions (3.8–4.6 overall severity), although the results of this testing are not included in this manuscript. The reduced toxicity of hydrothermal pretreatment has also contributed to our choice of these hydrothermal pretreatment methods.

Determination of pretreatment severity

1 $$\log \,R_{0} = t \times { \exp }\left( {\frac{(T - 100)}{14.75}} \right)$$

where log *R*_0_ is the severity factor, *t* is the residence time (min), and *T* is the pretreatment temperature (°C).

Cellulosic substrates are complex, and the characterization of the properties affecting enzymatic hydrolysis and fermentation are not well known [21, 22]. Enzymatic hydrolysis of glucan to glucose is a key step in the release of monomeric sugars for fermentation. Without efficient hydrolysis, high ethanol yields cannot be achieved with cellulosic substrates. Cellulose crystallinity has been shown to be a factor in enzymatic hydrolysis of substrates [23]. Maciel et al. [24] has shown C-13 nuclear magnetic resonance (NMR) imaging to be an effective method of determining the amount and crystal structure of sugars in substrates. In addition to crystallinity, Yoshida et al. [23] also showed that lignin content has an effect on enzymatic hydrolysis. Since the enzymes need access to the substrate to be effective, a substrate that falls out of solution will lower enzymatic access to the glycosidic linkages. In fact, mixing and viscosity should also have an effect on the enzymatic access of the substrate as the enzymes are suspended in solution and these factors both determine how readily these enzymes can come into contact with their targets [25]. Since the substrate is suspended in water, a polar solvent, the overall electrostatic charge of the substrate can also have an effect on the ability of the substrate to stay suspended. Ahmed and Labavitch [26] have shown that the biphenyl assay is a method for the enumeration of charge based on uronic acid content. Since cellulosic biomass consists of many different fractions including cellulose, hemicellulose, and lignin, a method to separate the cellulose fraction for further analysis would be beneficial. Pattathil et al. [27] have shown that performing a chlorite extraction on the substrate adequately separates the cellulose fraction from the residuals. Additional characterization of as many substrate components as possible was also needed to confirm the previous assays as well as to delve deeper into possible differences in the substrates. DeMartini et al. has also shown that glycome profiling can be a useful method to elucidate differences in specific residues in cellulosic substrates [27–30]. This method utilizes monoclonal antibodies (mAbs) with specificity to a particular bond or group.

Hydrothermal pretreatment functions to combat the recalcitrance of cellulosic feedstocks by using heat and pressure to break down the complex bonds between lignin, cellulose, and hemicellulose. A two-stage process using a lower severity factor for the first treatment step could potentially allow for a great recovery of the hemicellulose fraction as it should reduce the conversion of hemicellulose to furfural. This could lead to increased capture of the hemicellulose in the wash step between the two stages. The one-stage pretreatment would not allow for this enhanced hemicellulose recovery as the wash step occurs after the high severity pretreatment, although the wash step in both processes serves to remove inhibitors to fermentation. In our own work with hydrothermal pretreatment of woody feedstocks, we noted that even minor differences in pretreatment conditions could lead to very different outcomes during hydrolysis and fermentation. In this study, we will show that the ethanol yield during fermentation from two different hydrothermal pretreatment processes that have the same total pretreatment severity is very different. We will present a detailed analysis of the physical and chemical properties of these substrates using both traditional and recently developed methods, and propose how the differences observed could lead to the significant variation in fermentation performance.

## Results

**Fig. 1:**
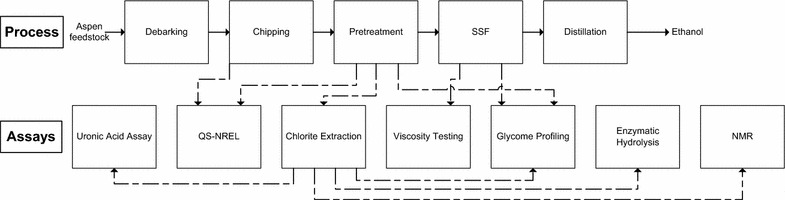
Diagram of the process and the additional assays performed. The *top row* indicates process steps while the *bottom row* indicates various assays and experiments performed. *Solid lines* indicate the flow of the process while *dashed lines* indicate where samples were taken to perform subsequent assays. Note that the pretreatment box is one element and contains one pretreatment step for MS1112 and two pretreatment steps for MS1107

**Fig. 2:**
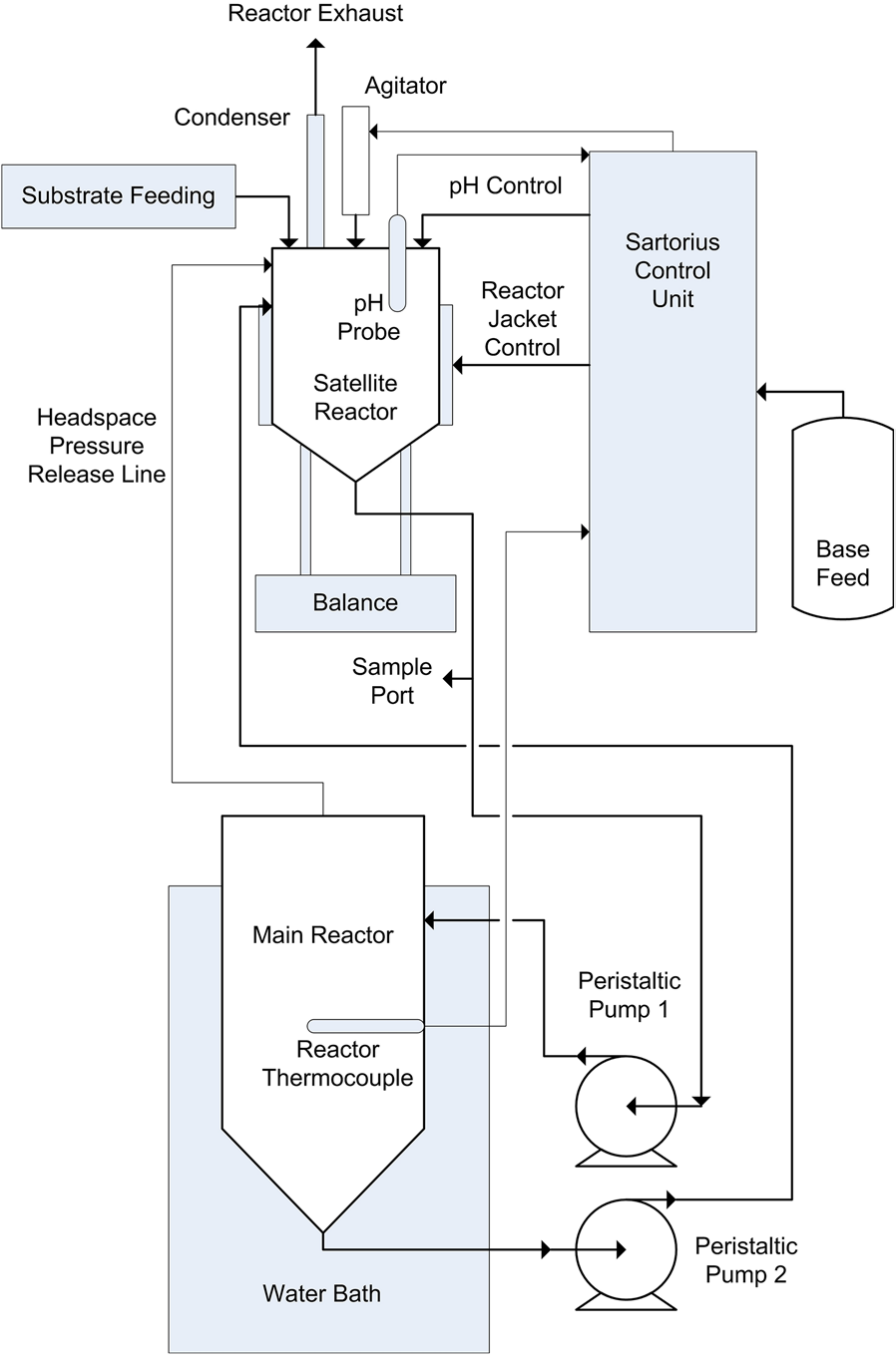
Diagram of 10 L reactor system highlighting the satellite reactor and the main reactor. Control and all monitoring except temperature are carried out in the satellite reactor. Peristaltic pump 1 was set to a specified flow rate and pump 2 was matched by a scale feedback loop to maintain the mass in the satellite reactor for a total main tank turnover rate of 3/h. Fermentations were carried out at 25 % TS, 800 rpm agitation in the satellite tank, pH 5, and 35 °C for 144 h with feeding lasting 60 h

Aspen chips (*Populus tremuloides*) were processed, pretreated, subjected to an SSF, and distilled while having various properties quantified (Fig. 1). Additionally, the aspen feedstock used to make these substrates was analyzed using QS and found to contain 50.76 ± 0.09 % cellulose and 22.2 ± 0.6 % hemicellulose. The chips were subjected to two separate pretreatment protocols, creating substrates MS1112 (one-stage pretreated hardwood), MS1107 (two-stage pretreated hardwood), and MS1105 (two-stage pretreated hardwood same conditions as MS1107 but a different batch number, hereby referred to as MS1107). When these substrates were fermented in a 2- or 10-L reactor, they produced very different results. A diagram of the 10 L fermentation system has been provided (Fig. 2).

### Fermentation

**Fig. 3:**
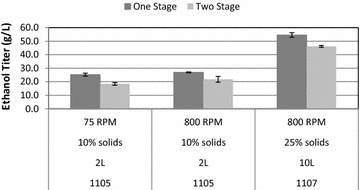
Fermentation results obtained via HPLC analysis after 144 h. The *black bars* represent the single-stage substrate and the *gray bars* represent the two-stage substrate. Fermentations were carried out at 35 °C and pH 5. At both the 75 and 800 rpm agitation rates, the fed batch fermentations on MS1112 resulted in higher ethanol titers than MS1105/7 at both the 2 and 10 L scale. Note the higher titers at increased agitation levels

**Fig. 4:**
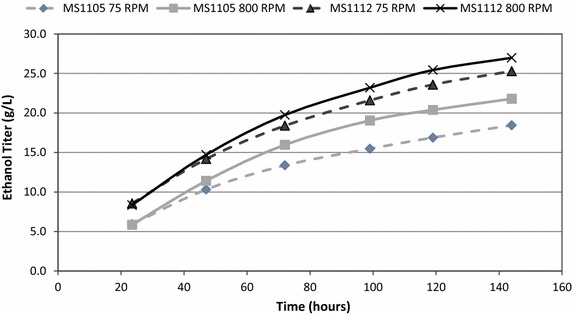
Time course data for 2 L fermentations. MS1112 exhibits a higher titer at all time points as does increased agitation for a given substrate. MS1112 is in *black* and MS1105 is in *gray*. *Dashed lines* indicate 75 rpm while *solid lines* indicate 800 rpm. Also note that the differences between two comparative fermentations increases as the fermentation progresses. These fermentations were carried out at 35 °C and pH 5 for 144 h

Fed batch fermentations carried out at both the 2 and 10 L scales resulted in higher final ethanol titers for MS1112 (Fig. 3). The final titers for MS1112 were as much as 37 % higher for the low solids, low mixing condition while it was still 18 % higher under the high solids, high mixing condition. MS1112 shows a higher increase under low mixing conditions than high mixing conditions as well as under high solids loading as compared to low solids loading. These increases were present throughout the duration of the fermentation as can be seen in the time course data (Fig. 4). The higher productivity of MS1112 continued to increase over the course of the fermentation even after the conclusion of feeding at 60 h. Using a method of accounting for the total sugar fed and total volume (Eq. 2), MS1112 showed an increase in yield over all the process conditions tested. Overall, titers were more than 18 % higher for MS1112, and ethanol yield on carbohydrate fed to the reactor was 9 % higher (Table 1). Using a theoretical ethanol yield of 0.48 g ethanol/g sugar loaded (slightly lower than the high value of 0.51 g/g), we see that the MS11112 fermentations reach between 65 and 80 % of the theoretical yield while the MS1107 fermentations reach only 60–71 % theoretical yield.

**Table 1:** Includes data on ethanol yield, QS, viscosity, settling, the uronic acid assay, and enzymatic hydrolysis

Substrate	MS1112	MS1105/MS1107
Parameter		
Ethanol yield		
2 L—75 rpm (w/w)	0.359	0.286
2 L—800 rpm (w/w)	0.384	0.340
10 L—800 rpm (w/w)	0.313	0.287
Quantitative saccharification (QS)		
Solids% cellulose (w/w)	63.88 ± 0.05	58.02 ± 0.03
Solids% hemicellulose (w/w)	0	7.90 ± 0.08
Solids% lignin (w/w)	28.0	33.0
Viscosity (cP)		
Raw (10 % solids)	420 ± 10	55 ± 5
Raw + ethanol (10 % solids)	545 ± 6	53 ± 5
Chlorite extraction (10 % solids)	27 ± 1	13.4 ± 0.01
Hydroxymethylfurfural (g/L)	74.55	84.00
Sedimentation volume%		
10 % solids, 10 min	91 ± 1	36 ± 1
10 % solids, 24 h	80 ± 2	35 ± 1
20 % solids, 10 min	99.9 ± 0.1	76 ± 1
20 % solids, 24 h	99.9 ± 0.1	65 ± 1
Uronic acid assay (relative absorbance)	24.75 ± 0.09	9 ± 3
Enzymatic hydrolysis (%)		
Pretreated hardwood	33.2	32.1
Chlorite-treated PHW	36.2	18.9

These results highlight the physical and chemical differences between the two substrates. MS1112 exhibits a higher ethanol yield and titer than MS1107 at all time points and conditions tested. MS1112 also exhibits increased sugar concentration, decreased lignin concentration, increased viscosity, increased settled volume, increased relative absorbance which indicates greater polarity, equivalent enzymatic sugar release with raw samples, and greater enzymatic sugar release with chlorite-pretreated samples

### QS, viscosity, and settling

MS1112 and MS1107 were characterized by a variety of assays in order to compare their physiochemical properties (Table 1). The quantitative saccharification shows that MS1112 contains a larger fraction of cellulose (63.88 ± 0.05 % compared to 54.97 ± 0.03 % w/w) than MS1107. This indicates that there is more sugar available in the MS1112 fermentations to start. The hemicellulose fraction present in MS1112 was lower than that found in MS1107 (0 % as compared to 7.90 ± 0.08 % (w/w). The QS results also indicate that there is a reduced amount of lignin in MS1112 compared to MS1107, 28.0 and 33.0 %, respectively, so not only is there more cellulose in the MS1112 fermentations, the reduced lignin content makes that cellulose more accessible to enzymatic digestion. The inhibitory hemicellulose is also removed at a higher rate in the MS1112 than the MS1107.

The two substrates also exhibited significantly different viscosities at a solids loading of 10 %. This held true for a 10 % solids loading in water alone, 420 ± 10 and 55 ± 5 cP for the single and two-stage, respectively. The much higher viscosity exhibited by MS1112 is indicative of differences in the interactions between the substrate and the solute. This result also held at a 10 % solids loading with 10 % of the water substituted for ethanol, 545 ± 6 and 53 ± 5 cP, respectively. The addition of the ethanol reducing the polarity of the solvent slightly to see if this yields a different result. A 10 % ethanol loading is also readily achieved during fermentation. The difference was no longer apparent once a chlorite extraction was performed on the two samples, 27 ± 1 and 13.4 ± 0.01 cP for the single- and two-stage substrates, respectively. Since the chlorite extraction serves to strip everything from the substrate leaving only cellulose, this result indicates that the differences are not likely caused by the cellulose structure itself.

There were also mixing issues during the fermentations completed with MS1112. It appeared much more viscous upon addition to the reactor vessels than MS1107 and would take longer to add to the reactor than MS1107 during each feeding which agrees with the above data. MS1112 also appeared fluffy and light before addition to the reactor as compared to MS1107 which appeared more sticky and granular with the presence of larger particles. MS1107 also required more careful addition of the larger particles to the 10 L reactor to prevent clogging of the lines connecting the main reactor vessel to the satellite vessel. MS1112 appeared to mix better after several hours in the reactor with MS1107 being prone to settling out of solution at lower agitation or upon stopping agitation.

Settling experiments indicate that the one-stage substrate occupies more volume (50 and 30 %) after 24 h at both 20 and 10 % solids loadings, respectively. After only 10 min, MS1112 already occupied 53 and 23 % more volume than MS1107 at the 20 and 10 % solids loadings, respectively. This highlights a large difference in the fundamental ability of the substrates to stay in solution and thereby promote enzymatic attack. Table 1 also shows that MS1112 exhibits a near 100 % settled volume at the 20 % solids loading which would indicate that this substrate is at or near its water saturation point at a 20 % solids loading. This corroborates the mixing issues that were encountered with this substrate during fermentation at a 25 % solids loading in that if the substrate is already occupying all the water holding capacity of the solution at 20 %, then addition of a further 5 % substrate is likely to result in increased viscosity and mixing issues.

The polarity of the two substrates was compared using a uronic acid assay. This assay measured the relative absorbance of multiple samples against a standard curve in order to determine the amount of uronic acids present. These results indicate that MS1112 exhibits a higher relative absorbance, 24.75 ± 0.09, than MS1107 9 ± 3. These results indicate that MS1112 has a higher polarity than MS1107 which could play a role in the quicker settling and lower viscosity exhibited by MS1107.

### Enzymatic digestion and NMR

Samples of both chlorite-extracted substrates were subjected to enzymatic hydrolysis using Flashzyme. The results show that the raw samples had a similar amount of carbohydrate released by enzymatic digestion. The chlorite-extracted samples showed drastic differences between the two samples with MS1112 having a similar amount of sugar released as was released by the raw samples, while MS1107 released approximately half of the sugar as the rest of the samples. This could indicate differences in the crystal structure of the cellulose in the two substrates.

**Fig. 5:**
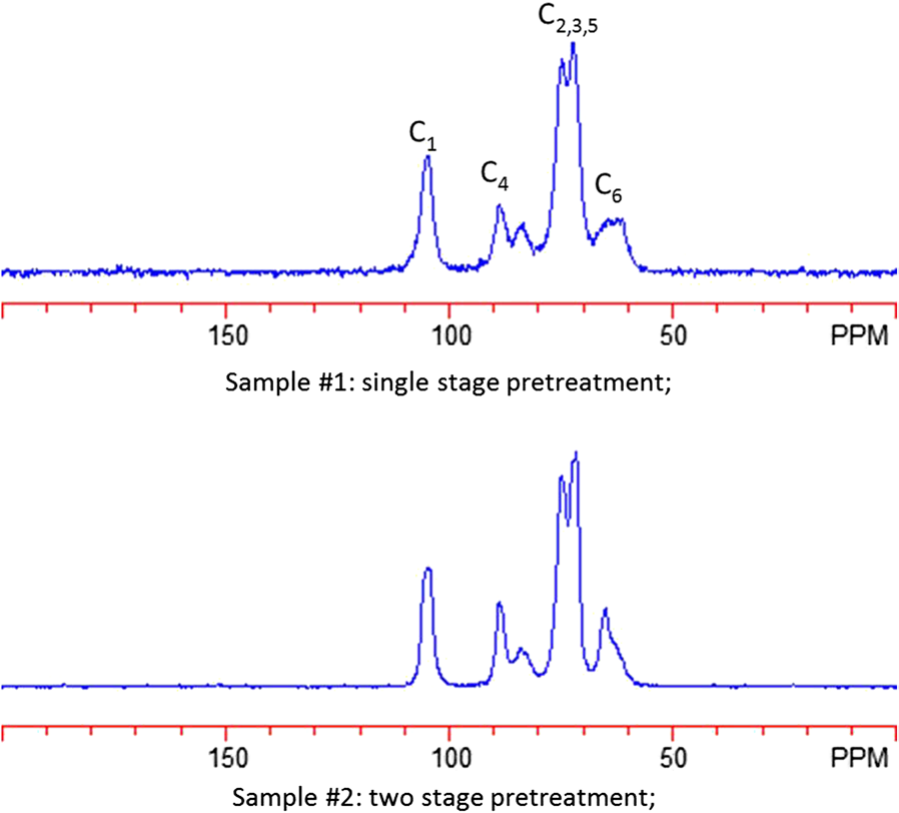
Solid-state CPMAS13 NMR data showing MS1112 versus MS1107. Here you can see that the C1–C5 residues appear similar with the only visible difference occurring in the C6 residue

To further elucidate the differences between these substrates, samples of chlorite-extracted substrate were subjected to analysis using CPMAS C^13^ NMR (Fig. 5). The results showed little differences between the samples at the C1–C5 carbon residues, which was expected. The analysis did indicate that there were some differences in the C6 residue which corresponds to residual glucose. It appears that MS1112 exhibits a 53.1 % crystallinity compared to 56.6 % crystallinity for MS1107. A higher crystallinity indicates that the substrate is less accessible to enzymatic attack.

### Glycome profiling

**Fig. 6:**
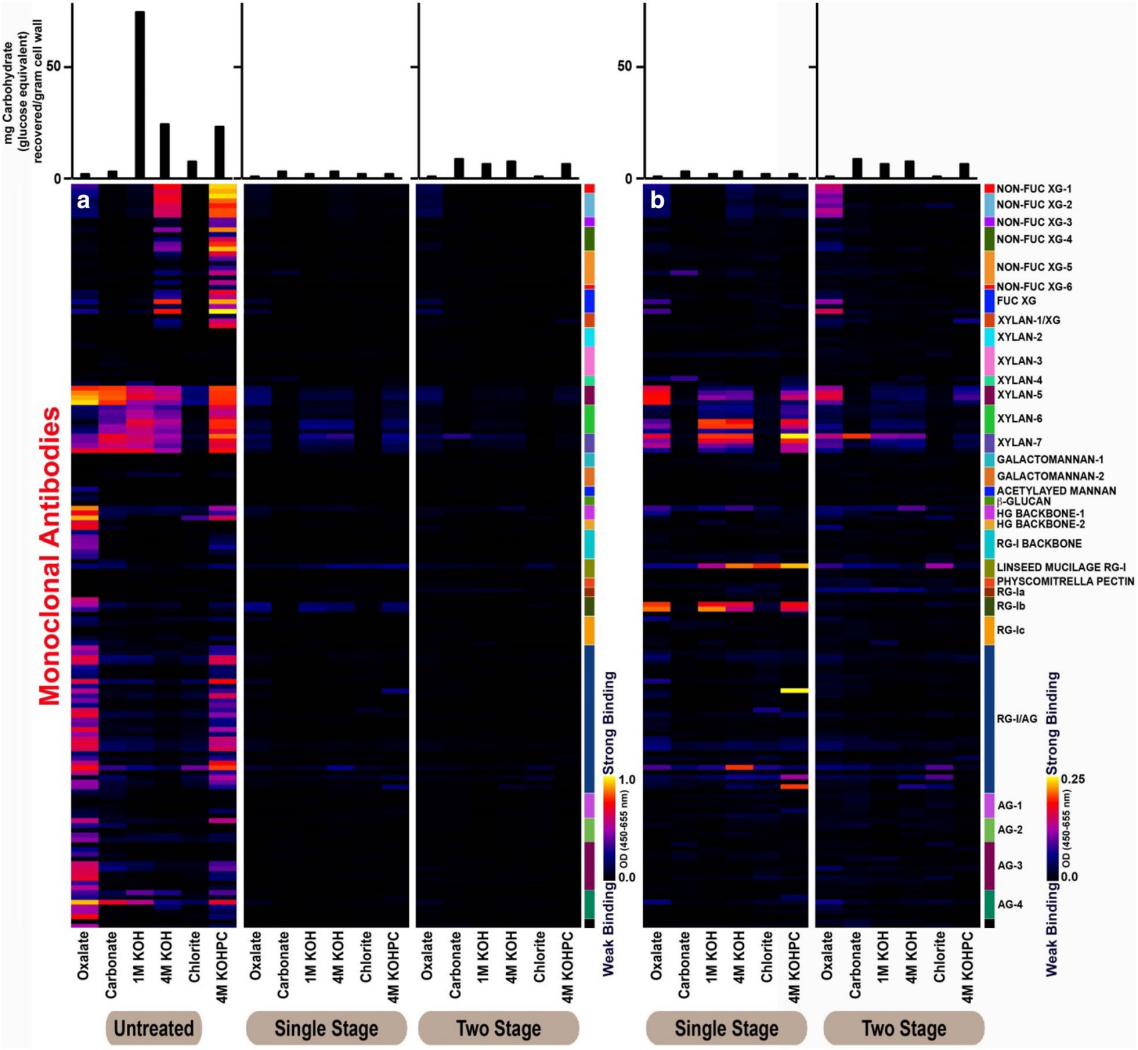
Glycome profiling analyses of untreated biomass, MS1112, and MS1107. Note the lack of non-cellulosic epitopes for both pretreated samples. Also of interest is the increased relative concentrations of pectic and hemicellulosic residues for the MS1112 substrate over the MS1107 substrate

Glycome profiling analyses of untreated biomass, MS1112, and MS1107 residues were conducted in order to elucidate overall changes in the non-cellulosic glycan structure, composition, and extractability (Fig. 6a, b). Glycome profiling of untreated aspen delineated the overall cell wall glycan composition of the samples (Fig. 6a). The least harsh extract, oxalate, contained essentially xylan, pectic backbone, and pectic arabinogalactan epitopes (indicated by the higher binding of xylan-5–xylan-7, HG-backbone-1, RG-I backbone, RG-I/AG, and AG-1–AG-4 groups of mAbs). Carbonate and 1 M KOH extracts contained mainly xylan epitopes. Major glycan epitopes detected in 4 M KOH extracts were those belonging to xylan and xyloglucans. Chlorite extracts containing glycan components that are released upon lignin fragmentation exhibited only trace abundance of xylan and pectic arabinogalactan epitopes. The extract made under the most severe conditions, 4 M KOHPC extract, showed significant abundance of xylan, xyloglucans, pectic backbone, and pectic arabinogalactan epitopes (Fig. 6a).

Sequential extractions (see bottom panel) were made using increasingly harsh reagents from cell wall isolates of various biomass materials as explained in the materials and methods. The extracts were subsequently screened with a comprehensive suite of cell wall glycan-directed mAbs that recognize most major classes of non-cellulosic glycans present in plant cell walls (see panel on the right). The binding intensities of mAbs are depicted as heatmaps with bright yellow, red, and dark blue colors. Bright yellow and dark blue are depicting the maximum and minimum binding, respectively. Three panels on the left (a) are scaled 0–1 for the optical density values (binding strength of mAbs) and two panels on the right (b) represent the same data of one-stage and two-stage samples from a, but represented in a scale of 0–0.25. The carbohydrate amounts recovered (glucose equivalents) in each extracts are shown in the top bar graphs.

Glycome profiles of both MS1112 and MS1107 biomass residues were significantly different from that of untreated material (Fig. 6a). A significant reduction in the abundance of almost all non-cellulosic glycan epitopes was evident in the glycome profiles of both pretreated materials (Fig. 6a second and third profile panels). This was also reflected in the amounts of carbohydrate recovered (measured in glucose equivalents, see upper bar graphs) in cell wall extracts. As expected under the pretreatment used, almost all non-cellulosic glycan epitopes were removed in both materials. Similar results were reported previously on hydrothermally pretreated poplar biomass [28]. Significantly reduced amounts of non-cellulosic glycan epitopes were apparent in the glycome profiles of MS1112 and MS1107 pretreated biomass, as indicated by the reduced binding intensities of mAbs in their heatmaps (Fig. 6a). Differences between MS1112 and MS1107 were of interest. A better visualization of abundances of these glycan epitopes was made possible by visualizing the heatmaps of just the pretreated samples by reducing the maximum value of the optical density scale (that correspond to the binding intensities of mAbs) to 0.25 which was the highest binding value in the pretreated samples (note that in Fig. 6a, this maximum value is 1.0). These corresponding scale changed heat maps are depicted in Fig. 6b. There were differences in the residual amounts of glycan epitopes between glycome profiles of MS1112 and MS1107. A higher proportion of xylan epitopes were observed in MS112 in the oxalate, 1 M KOH, 4 M KOH, and 4 M KOHPC extracts. This indicates the presence of a relatively higher proportion of residual hemicelluloses in MS1112. Additionally, several pectic epitopes, especially those recognized by certain pectin-specific mAbs were also more abundant in MS1112. Overall, these data show that the walls of MS1112 and MS1107 differed in their structures and composition which could explain some of the differences seen in fermentation.

## Discussion

The Aspen substrate showed a high fraction of cellulose and hemicellulose when compared to other feedstocks. This is comparable to 37 % cellulose and 24 % hemicellulose for rice straw [31] and 45.2 ± 1.81 % cellulose and 26.6 ± 1.23 % for *Parthenium hysterophorus* [32]. The higher cellulose content agrees with the increased ethanol titers and increased yields seen in the fermentations of MS1112. The greater removal of the hemicellulose fraction in the MS1112 is a useful reduction in inhibitors although it is interesting. We would expect the extra pretreatment step that the MS1107 undergoes to remove more of the hemicellulose. This could potentially be a function of the structure of the lignin changing due to the multiple heat and pressure steps causing a different interaction with the hemicellulose fraction causing the MS1107 to retain more hemicellulose as well as more lignin. The potential for lignin and hemicellulose changes here should be further investigated.

While hardwood feedstocks are potentially more recalcitrant, the higher sugar concentration makes them a worthwhile target for pretreatment and fermentation. Our studies showed that conversion of MS1112 during fermentation was better than conversion of MS1107. Conversion for MS1112 was more rapid and resulted in higher ethanol titers and yields, indicating differences in either the quantity of cellulose that is available for fermentation, the structure of the cellulose present, physical differences in the substrates, or chemical differences in the substrates. The fermentation profiles (Fig. 4) show that the fermentation rates are similar but appear to start lower for both examples of the MS1107. This could indicate that the cellulose is less accessible in the two-stage substrate than the single-stage. It could be a function of the enzymes taking a longer time to begin freeing up the cellulose.

The levels of cellulose, hemicellulose, and lignin present in the pretreated aspen substrates are comparable to the levels found during acid pretreatment of *P. hysterophorus* which are 64.1 ± 2.5 % for cellulose, 1.02 ± 0.073 % for hemicellulose, and 31.6 ± 1.04 % for lignin (w/w) [32]. The results indicate that there are lower levels of cellulose present in MS1107 than in MS1112 which agrees with the fermentation results that show lower ethanol production at all time points. This could be caused by a higher level of degradation in MS1107 of cellulose to by-products including HMF during pretreatment. Increased heat and pressure are known to degrade sugars more than lower parameters so while the overall severity is the same for the two substrates; it is possible that subjecting MS1107 to additional heat and pressure further degraded the cellulose [33]. Unfortunately, much of the HMF is off gassed during the pretreatment process making quantification difficult, although the HPLC results do show that MS1107 has slightly higher levels of HMF present. This could render the one-stage process a more viable option, as the additional pretreatment step in MS1107 would increase the cost of the process while also releasing less carbohydrate.

A higher initial cellulose concentration does not appear to be the only difference between the substrates as shown by the ethanol yield data that corrects for the amount of sugar loaded and compare the fermentations to theoretical values. The fact that differences in ethanol titer are seen almost as soon as the fermentation begins would indicate that there is something additional going on with the release of the monomeric sugars from the substrate. It is possible that there is something different with the cellulose structure of the two substrates. The higher crystallinity exhibited by MS1107, seen in the NMR results, has been shown to inhibit enzymatic attack [34]. The reduced sugar release during the enzymatic hydrolysis of the chlorite-pretreated substrates could also be indicative of this issue. The enzymatic hydrolysis data appear to refute this theory though as both samples appeared to release a similar amount of glucose, however the enzymatic hydrolysis was run at a 2.5 % TS. This could indicate that the solubility of both substrates is higher under the lower solids loading. The enzymatic hydrolysis also failed to release approximately two-thirds of the available sugar possibly indicating that the readily available substrate was all that was released. Additionally, the higher solids loading in the fermentation could give rise to increased lignin inhibition and also charge issues under the increased loading. This could speak of the physical and chemical differences in the substrate playing a larger role than the actual crystalline structure of the cellulose or just the limitations of the enzymatic hydrolysis as performed. Allowing the enzymatic hydrolysis to run longer could have given insight into this issue.

The large number of physical and chemical differences between the two substrates indicate that the two pretreatment methods are having significant effects on the substrate. The decreased lignin present in MS1112 was an unexpected finding. The hypothesis proposed was that since both substrates were pretreated to the same overall severity, the lignin content would be similar. Mosier et al. has shown that the lower lignin content in MS1112 could indicate that the cellulose is more available to enzymatic digestion since lignin has been shown to lower enzyme effectiveness [15]. It is also possible that the lignin has been condensed and is coating the substrate in MS1107. This would also cause the cellulose to be more inaccessible to the enzymes which could contribute to the lower ethanol titers and yields, especially when compared to theoretical values, seen in MS1107 fermentations [9, 14]. While it appears that the lignin is a higher percentage of the overall makeup of MS1107, it is possible that the lignin is degraded but other components are also degraded, thereby effectively increasing the relative lignin content. This is corroborated by the fact that the cellulose fraction in MS1107 is lower thereby indicating that the increased lignin content could be an artifact of cellulose degradation.

The differences in appearance between the two substrates (MS1112 being light and fluffy and going into solution readily while MS1107 is dense and compact and falls out of solution easily) could be an indication that the second-stage of pretreatment changed some structure or complex in the substrate which renders the carbohydrate less accessible than in the one-stage substrate [35]. This phenomenon could be compounded by the differences in viscosity. While the higher viscosity of MS1112 has caused issues with mixing, it also allows the solution to stay homogeneous. The lack of viscosity differences when the substrates are subjected to a chlorite extraction lend more weight to their being something different in the overall structure of the substrate as the extraction strips all but the cellulose [27, 36]. This indicates that the differences seen have more to do with the overall structure and the makeup of the substrate as opposed to just the crystalline cellulose. This coupled with the fact that MS1112 does not settle out of solution as readily as MS11707 could reinforce the possibility that MS1107 is not as readily digestible as MS1112 due to physical and chemical changes. The first step in hydrolysis is ensuring the enzymes have ready access to their specific residues. The uronic acid assay results further bolster this argument since the higher charge present in MS1112 would indicate that the substrate exhibits a higher polarity than MS1107 which indicates it will stay in an aqueous solution better [37]. The above observations on lignin also corroborate this theory as lignin is hydrophobic. This goes hand-in-hand with the observations of viscosity, settling, and mixing.

Overall, the pattern of glycan extractability in untreated aspen feedstock used in this study, as indicated by glycome profiling (Fig. 6a), was mostly similar to previously reported data on untreated poplar biomass samples [28]. Glycome profiling studies showed that there was a significant removal of non-cellulosic glycan epitopes in both MS1112 and MS1107 compared to untreated biomass material. Again, MS1107 showed a higher degree of non-cellulosic glycan epitope removal in comparison to MS1112 (Fig. 6b). Previous studies have reported this effect of hydrothermal pretreatment on poplar biomass [28]. This significantly reduced binding of mAbs to cell wall extracts from pretreated materials is potentially due to either mass removal of non-cellulosic glycan epitope structures induced by pretreatment conditions or due to significant shortening of glycans caused by pretreatment induced fragmentation [28]. It is interesting that MS1112, with a higher proportion of non-cellulosic glycan epitopes, exhibited higher conversion and ethanol production than MS1107 where nearly all non-cellulosic epitopes were removed. This is potentially due to the effect of two-stage pretreatment conditions causing increased crystallinity of cellulose, increased conversion of cellulose to HMF, as well as the physical and chemical changes discussed in the previous paragraphs. The presence of pectin and xylan epitopes after one-stage pretreatment is likely due to the presence of tightly wall-integrated classes of pectins, potentially through their strong associations with other wall components including hemicellulose and lignin, which are not removed during the single-stage pretreatment process. Previous studies with poplar biomass have demonstrated the presence of tightly integrated pectic components potentially integrated into the cell wall structure through associations with hemicelluloses and lignin [28]. The thought here is that the additional pretreatment step further degraded the pectin and hemicellulose fractions that were recalcitrant and were not able to be washed out in the washing step, however, more studies are needed to elucidate this complex mechanism.

## Conclusions

The results from this study indicate that one-stage hydrothermal pretreated material performs better during fermentation that two-stage hydrothermal pretreated material under the conditions tested here. Additional fermentations were performed on one- and two-stage hot water pretreated hardwoods ranging from a severity of 3.8 to 4.6 with similar results (data not shown). The single-stage pretreatment resulted in higher sugar concentrations, decreased lignin concentrations, reduced cellulose crystallinity, higher ethanol titers, and higher ethanol yield. The physical and chemical properties outlined above are more favorable to the production of cellulosic ethanol, namely an increase in viscosity, an increased settled volume, increased polarity, and a reduction in degradation of glucose to HMF. These factors are believed to allow for better enzymatic accessibility. However, the two-stage process allows for enhanced recovery of the hemicellulose stream. If this stream is fermented as well, the overall process yield should increase. For this reason, it can not be said definitively that the one-stage process is better. The results from this study, indicating certain advantageous properties of one-stage material, should be applied to the two-stage process to optimize the pretreatment process in order to maximize the overall yield. The combination of the favorable properties of the single-stage substrate with the hemicellulose extraction and fermentation available from the two-stage substrate has the potential to push this cellulosic technology into closer competition with traditional fossil fuels.

## Methods

### Materials

All reagents and chemicals were obtained from Sigma-Aldrich with the following exceptions:

- Flashzyme—AB Enzymes;
- Aspen chips sourced from a lumber mill in Alberta, Canada.

### Feedstock/pretreatment

The initial feedstock used in these experiments was Aspen sourced from Canada which was fed to the pretreatment process at a %TS of 41.8 ± 3.8 %. The whole logs were debarked and tub ground to yield an optimal size of 1″ × 1″ × 3/16″. The maximum allowable thickness was set to ¼″ while the maximum length or width was set to 1.5″ to reduce equipment issues during the process. These chips were delivered to Mascoma’s Rome, NY pilot plant facility. It is well known that different forms of pretreatment yield different results for hardwood cellulosic fermentations [11]. These include AFEX™ chemical, mechanical, and thermal pretreatment among others. Two forms of hydrothermal pretreatment were utilized. The first method resulted in MS1112 where raw Aspen chips were processed in a horizontal ANDRITZ digester at 205 °C and 235 psig for 20.5 min resulting in a severity of 4.40. The resulting chips are then steam exploded through a 1/8″ orifice before being sent to a hot water wash step for 3 h at 87.8 °C. The hot water wash step was carried out to remove the hemicellulose stream which can be fermented as well. The resulting solid stream was then centrifuged and allowed to floor dry until it reached 63 % TS. MS1107 differed in that there is an additional pretreatment step. The raw Aspen chips were first passed through the ANDRITZ digester at 187 °C and 155.7 psig for 25 min resulting in a severity of 3.96. The chips were then steam exploded as before, washed to remove the hemicellulose for 3 h at 87.8 °C, and allowed to air dry. The additional pretreatment step was carried out after the floor drying. The dry fiber was passed through the ANDRITZ digester a second time at 210 °C and 262 psig for 15 min for a final severity of 4.41. The wood was then steam exploded a second time and allowed to dry. The major difference between the two processes is that the first pass through the ANDRITZ digester occurs at a lower temperature and pressure resulting in less degradation of the valuable hemicellulose stream. This means that the yield in the two-stage process is higher, but also that the pretreatment process is more energy and time intensive than the one-stage process.

### Fermentation

This fiber was then subjected to a simultaneous saccharification and fermentation (SSF) procedure that lasts 144 h for the purposes of digesting the complex carbohydrates in the substrate into simple sugars which are then fermented to ethanol. These fermentations were carried out at both the 2 and 10 L scales. The substrate was fed over the first 60 h of the fermentation to a satellite tank with overhead agitation in the 10 L scale. The fermentation contains 25 % TS at the end of feeding. The mixed slurry was then pumped over to the main tank. Agitation of the fermentation was achieved by the constant pumping between the satellite tank and the main tank. This occurred at a rate of three main tank turnovers per hour. The satellite tank was also equipped for controlling temperature and pH control. These tanks were kept at 35 °C and a pH of 5.0, respectively. 12 g/L corn steep liquor, 0.5 g/L diammonium phosphate, 0.1 mL/kg penicillin, and 5 mg enzyme/g TS were initially added to the fermentations. 15 M ammonium hydroxide was used for pH control. The 2-L fermentations were carried out in the same manner as the 10-L fermentations except there was only one reaction vessel with overhead agitation that contained all process controls. Samples were taken starting at 24 h and approximately every 24 h for the duration of the fermentations.

### Ethanol yield

Ethanol yield for both MS1112 and MS1107 was determined using 144 h fed batch fermentations at both the 2 and 10 L scales along with the total sugars in each fermentation and is calculated in Eq. 2.

The determination of ethanol yield in mass of ethanol produced per mass of sugar loaded. Ethanol titer is determined via HPLC analysis, fermentation volume is measured at the end of fermentation, biomass sugar content is taken via QS and HPLC, and the total dry substrate loaded is based on oven dry solids and total mass

2 $$\begin{aligned} & {\text{Ethanol}}\,{\text{Yield}} \\ & \quad = \frac{{{\text{Ethanol}}\,{\text{Titer}}\,\left( {\frac{\text{g}}{\text{L}}} \right) \times {\text{Fermentation}}\,{\text{Volume}}\,({\text{L}})}}{{{\text{Biomass }}\,{\text{Sugar}}\,{\text{Content}}\left( {\frac{\text{g}}{\text{g}}} \right) \times {\text{Total }}\,{\text{Dry}}\,{\text{Substrate}}\,{\text{Loaded}}\,({\text{g}})}} \\ \end{aligned}$$

The ethanol titer is determined using HPLC analysis of the resulting fermentation broth, fermentation volume is determined from the total volume loaded into the reactor, biomass sugar content is determined from QS analysis of the substrate and the total dry substrate loaded is a known quantity by mass.

### Yeast

A proprietary genetically engineered yeast strain supplied by Mascoma LLC was used in these experiments. This yeast was not genetically engineered to provide additional enzymatic digestion of the substrate but it was optimized for the SSF process.

### Quantitative saccharification (QS)

This assay was used to determine the total sugars and lignin present in the raw wood chips [38], the pretreated biomass, and the fermentation residuals. The procedure followed is outlined in the National Renewable Energy Laboratory (NREL) technical report NREL/TP-510-42618 [39].

### High performance liquid chromatography (HPLC)

HPLC was used to determine solute concentrations for carbohydrates as well as ethanol following the NREL protocol from technical report NREL/TP-510-42623 [40].

### Viscosity

Each sample was dried in an oven overnight at 50 °C to determine the total solids of the sample. The samples then had water added to them to bring the total solids to 10 % w/w. An additional raw sample was also prepared with 10 % of the water replaced with ethanol to determine if reducing the polarity of the liquid would yield any differences in viscosity.

### Sedimentation volume assay

The settling experiment was carried out by adding MS1112 and MS1107 to separate 1 L beakers of DI water until a 20 % w/w concentration was reached at a total mass of 500 g. This step was then repeated except these beakers were brought to a 10 % w/w concentration and a total mass of 500 g. The beakers were then stirred with a stir bar for 10 min at 400 rpm. The slurry was then poured into a 500 mL graduated cylinder and the volume of settled fiber was recorded at 10 min and 24 h.

### Biphenyl assay for uronic acids

The uronic acid assay was used to determine relative levels of galacturonic acid and glucuronic acid in the two substrates. Please see the Additional file 1 for the protocol.

### Chlorite extraction

The chlorite extraction was performed as described on page 64 of *Biomass Conversion*, 2012, Pattathil et al. [27, 36].

### Enzymatic hydrolysis of cellulose

To determine the accessibility of the substrate to enzymatic attack, a hydrolysis experiment was done in which a dose curve of commercially available enzyme, Flashzyme from AB Enzymes, was added to 2.5 % substrate (either pretreated or chlorite extracted pretreated hardwood) diluted in 50 mM sodium citrate buffer, pH 5.2. The plate was incubated at 35 °C while stirring and sampled daily for carbohydrate analysis by HPLC on a BioRad 87H column as described in (method below). The amount of sugar released enzymatically was compared to the values obtained by quantitative saccharification to determine the extent of hydrolysis.

### Solid-state CPMAS C^13^ NMR characterization

The solid-state CP/MAS C^13^ NMR experiments were performed to determine differences in cellulose structure [24, 41] on a Bruker Avance III 400 MHz spectrometer operating at frequencies of 100.59 MHz for C^13^ using a Bruker double-resonance 4-mm MAS probe head at ambient temperature. The samples were packed in a 4-mm ZrO rotor fitted with a Kel-F cap and spun at 8000 Hz. CP/MAS C^13^ data were acquired with a Bruker CP pulse sequence with pulse delay of 4 s, contact pulse of 2000 ms, and 2048 number of scans. Each sample was run duplicate and crystallinity results were averaged.

### Glycome profiling

Glycome profiling of untreated and various pretreated biomass residues that involve the preparation of sequential cell wall extracts and their Enzyme-Linked Immunosorbent Assay (ELISA-based mAb screenings) were carried out as previously described [27, 28].

Plant cell wall glycan-directed mAbs were from laboratory stocks (CCRC, JIM and MAC series) at the Complex Carbohydrate Research Center (available through CarboSource Services; http://www.carbosource.net) or were obtained from BioSupplies (Australia) (BG1, LAMP). Supporting information on mAbs [29] used in this study can be found in the Additional file 1: Table S1, including the link to WallMabDB (http://www.wallmabdb.net) that provides detailed information for each antibody.

### Future work

Since the data show that there are fundamental physical and chemical differences between the two substrates, a logical future line of questioning would revolve around optimizing the pretreatment parameters to increase to overall process yield. Finding a way to balance the economic advantages of MS1112 with the high yield of MS1107 would result in the most economically beneficial situation. This would require optimizing pretreatment time and temperature since the data show that using the simple severity parameter can be misleading. Another line of questioning revolves around nailing down why MS1107 is so much different from MS1112. This is really a process design question. Is it the second pass through the ANDRITZ unit that changes things so drastically or is it the second steam explosion step? Could it be that, even though the final severity (4.4) is the same for both substrates, the process of a second hydrothermal treatment changes the physical and chemical properties of the substrate? The lab scale system would be ideal for answering some of these fundamental questions and performing experiments to find more favorable process parameters. For example, two-stage pretreated material generated without the second steam explosion step could be fermented to parse out the effects the second steam explosion has on fermentation.

## Additional file

10.1186/s13068-016-0446-2 Biphenyl assay for uronic acids protocol and listing of monoclonal antibody names and their corresponding recognized glycan groups.

## Abbreviations

AFEX™: Ammonia Fiber Expansion
BESC: Bioenergy Science Center
CCRC: Complex Carbohydrate Research Center
CPMAS C^13^ NMR: cross polarized magic angle spinning carbon 13 nuclear magnetic resonance spectroscopy
ELISA: enzyme-linked immunosorbent assay
HMF: hydroxymethylfurfural
HPLC: high pressure liquid chromatography
KOH: potassium hydroxide
mAbs: monoclonal antibodies
MAS: magic angle spinning
MHz: mega hertz
NMR: nuclear magnetic resonance
NREL: National Renewable Energy Laboratory
psig: pounds per square inch gage
QS: quantitative saccharification
rpm: revolutions per minute
SSF: simultaneous saccharification and fermentation
TS: total solids
