# Supplementary material for: Physical and chemical differences between one-stage and two-stage hydrothermal pretreated hardwood substrates for use in cellulosic ethanol production
Source: Biotechnol Biofuels. 2016 Feb 3;9:30. doi: 10.1186/s13068-016-0446-9 (PMC4741017; doi:10.1186/s13068-016-0446-9)
Supplement: Supplementary file 1 — 10.1186/s13068-016-0446-2 Biphenyl assay for uronic acids protocol and listing of monoclonal antibody names and their corresponding recognized glycan groups. [file 13068_2016_446_MOESM1_ESM.docx]

**Biphenyl Assay for Uronic acids**

**Reagents**

0.0125M Sodium Borate reagent – 238.5mg Na2B4O7(10H2O) to 50ml concentrated H2SO4

0.15% m-hydroxy-biphenyl reagent *(in 5% NaOH) - 100ul of 50% NaOH solution diluted to 10ml,

15mg m-hydroxy-phenol to 10ml in 5% NaOH

Galacturonic acid in water (1mg/ml)

Glucuronic acid in water (1mg/ml)

*m-hydroxy-biphenyl=3-phenylphenol

**Equipment**

Boiling water bath

Pipettes

microtitre plates and reader

**Standards:**

1. Create standard curves as follows:

GlcA (1ug/ul)

0ul

5ul

10ul

20ul

50ul

Add deionized water so that the standard volume is 100ul in each tube.

2. Add 600ul of Borate reagent to each tube.

3. Heat in boiling water bath for 5 minutes.

4. Remove and let cool to room temperature (about 15 min.).

5. Add 20ul of biphenyl reagent to each tube.

6. Mix well and wait 20 min.

7. Transfer 150ul from each tube into a well in a 96 well plate. Use a positive displacement pipette and use the map below.

8. Read at abs 540.

**Samples:**

1. Run samples with 0, 20, 40, and 80ul of sample. Run in duplicate.

2. Adjust total sample volume to 100ul with deionized water.

3. Add 600ul of Borate reagent to each tube.

4. Heat in boiling water bath for 5 minutes.

5. Remove and let cool to room temperature (about 15 min.).

6. Add 20ul of biphenyl reagent to each tube.

7. Mix well and wait 20 min.

8. Transfer 150ul from each tube into a well in a 96 well plate. Use a positive displacement pipette and use the map below.

9. Read at abs 540.

**Supplementary Table S1:** Detailed list of cell wall glycan-directed monoclonal antibodies (mAbs) used for glycome profiling analyses. The groups of antibodies are based on a hierarchical clustering of ELISA data generated from a screen of all mAbs against a comprehensive panel of plant polysaccharide preparations (Pattathil et al., 2010; Pattathil et al., 2012) that clusters mAbs according to the predominant polysaccharides that they recognize. The majority of listings link to the Wall*Mab*DB plant cell wall monoclonal antibody database (http://www.wallmabdb.net) that provides detailed descriptions of each mAb, including immunogen, antibody isotype, epitope structure (to the extent known), supplier information, and related literature citations.

**Glycan Group Recognized mAb Names**

| Non-Fucosylated Xyloglucan-1 | CCRC-M95 |
| --- | --- |
|  | CCRC-M101 |
|  |  |
|  |  |
| Non-Fucosylated Xyloglucan-2 | CCRC-M104 |
|  | CCRC-M89 |
|  | CCRC-M93 |
|  | CCRC-M87 |
|  | CCRC-M88 |
|  |  |
|  |  |
| Non-Fucosylated Xyloglucan-3 | CCRC-M100 |
|  | CCRC-M103 |
|  |  |
|  |  |
| Non-Fucosylated Xyloglucan-4 | CCRC-M58 |
|  | CCRC-M86 |
|  | CCRC-M55 |
|  | CCRC-M52 |
|  | CCRC-M99 |
|  |  |
|  |  |
| Non-Fucosylated Xyloglucan-5 | CCRC-M54 |
|  | CCRC-M48 |
|  | CCRC-M49 |
|  | CCRC-M96 |
|  | CCRC-M50 |
|  | CCRC-M51 |
|  | CCRC-M53 |
|  |  |
|  |  |
| Non-Fucosylated Xyloglucan-6 | CCRC-M57 |
|  |  |
|  |  |
| Fucosylated Xyloglucan | CCRC-M102 |
|  | CCRC-M39 |
|  | CCRC-M106 |
|  | CCRC-M84 |
|  | CCRC-M1 |
|  |  |
|  |  |
| Xylan-1/XG | CCRC-M111 |
|  | CCRC-M108 |
|  | CCRC-M109 |
|  |  |
|  |  |
| Xylan-2 | CCRC-M119 |
|  | CCRC-M115 |
|  | CCRC-M110 |
|  | CCRC-M105 |
|  |  |
|  |  |
| Xylan-3 | CCRC-M117 |
|  | CCRC-M113 |
|  | CCRC-M120 |
|  | CCRC-M118 |
|  | CCRC-M116 |
|  | CCRC-M114 |
|  |  |
| Xylan-4 | CCRC-M154 |
|  | CCRC-M150 |
|  |  |
|  |  |
| Xylan-5 | CCRC-M144 |
|  | CCRC-M146 |
|  | CCRC-M145 |
|  | CCRC-M155 |
|  |  |
|  |  |
| Xylan-6 | CCRC-M153 |
|  | CCRC-M151 |
|  | CCRC-M148 |
|  | CCRC-M140 |
|  | CCRC-M139 |
|  | CCRC-M138 |
|  |  |
|  |  |
| Xylan-7 | CCRC-M160 |
|  | CCRC-M137 |
|  | CCRC-M152 |
|  | CCRC-M149 |
|  |  |
|  |  |
| Galactomannan-1 | CCRC-M75 |
|  | CCRC-M70 |
|  | CCRC-M74 |
|  |  |
| Galactomannan-2 | CCRC-M166 |
|  | CCRC-M168 |
|  | CCRC-M174 |
|  | CCRC-M175 |
|  |  |
|  |  |
| Glucomannan | CCRC-M169 |
|  | CCRC-M170 |
|  |  |
|  |  |
| β-Glucan | LAMP |
|  | BG1 |
|  |  |
|  |  |
| HG Backbone-1 | CCRC-M131 |
|  | CCRC-M38 |
|  | JIM5 |
|  |  |
|  |  |
| HG Backbone-2 | JIM136 |
|  | JIM7 |
|  |  |
|  |  |
| RG-I Backbone | CCRC-M69 |
|  | CCRC-M35 |
|  | CCRC-M36 |
|  | CCRC-M14 |
|  | CCRC-M129 |
|  | CCRC-M72 |
|  |  |
|  |  |
| Linseed Mucilage RG-I | JIM3 |
|  | CCRC-M40 |
|  | CCRC-M161 |
|  | CCRC-M164 |
|  |  |
|  |  |
| Physcomitrella Pectin | CCRC-M98 |
|  | CCRC-M94 |
|  |  |
|  |  |
| RG-Ia | CCRC-M5 |
|  | CCRC-M2 |
|  |  |
|  |  |
| RG-Ib | JIM137 |
|  | JIM101 |
|  | CCRC-M61 |
|  | CCRC-M30 |
|  |  |
|  |  |
| RG-Ic | CCRC-M23 |
|  | CCRC-M17 |
|  | CCRC-M19 |
|  | CCRC-M18 |
|  | CCRC-M56 |
|  | CCRC-M16 |
|  |  |
|  |  |
| RG-I/Arabinogalactan | CCRC-M60 |
|  | CCRC-M41 |
|  | CCRC-M80 |
|  | CCRC-M79 |
|  | CCRC-M44 |
|  | CCRC-M33 |
|  | CCRC-M32 |
|  | CCRC-M13 |
|  | CCRC-M42 |
|  | CCRC-M24 |
|  | CCRC-M12 |
|  | CCRC-M7 |
|  | CCRC-M77 |
|  | CCRC-M25 |
|  | CCRC-M9 |
|  | CCRC-M128 |
|  | CCRC-M126 |
|  | CCRC-M134 |
|  | CCRC-M125 |
|  | CCRC-M123 |
|  | CCRC-M122 |
|  | CCRC-M121 |
|  | CCRC-M112 |
|  | CCRC-M21 |
|  | JIM131 |
|  | CCRC-M22 |
|  | JIM132 |
|  | JIM1 |
|  | CCRC-M15 |
|  | CCRC-M8 |
|  | JIM16 |
|  |  |
|  |  |
| Arabinogalactan-1 | JIM93 |
|  | JIM94 |
|  | JIM11 |
|  | MAC204 |
|  | JIM20 |
|  |  |
|  |  |
| Arabinogalactan-2 | JIM14 |
|  | JIM19 |
|  | JIM12 |
|  | CCRC-M133 |
|  | CCRC-M107 |
|  |  |
|  |  |
| Arabinogalactan-3 | JIM4 |
|  | CCRC-M31 |
|  | JIM17 |
|  | CCRC-M26 |
|  | JIM15 |
|  | JIM8 |
|  | CCRC-M85 |
|  | CCRC-M81 |
|  | MAC266 |
|  | PN 16.4B4 |
|  |  |
|  |  |
| Arabinogalactan-4 | MAC207 |
|  | JIM133 |
|  | JIM13 |
|  | CCRC-M92 |
|  | CCRC-M91 |
|  | CCRC-M78 |
|  |  |
|  |  |
| Unidentified | MAC265 |
|  | CCRC-M97 |
|  |  |
